# The LITA–LAD coronary bypass: historical perfection with emerging future perspectives in the third millennium

**DOI:** 10.3389/fcvm.2026.1749380

**Published:** 2026-01-29

**Authors:** Dritan Useini, Ingo Kutschka, Hassina Baraki

**Affiliations:** Department of Cardiac Surgery, University Hospital Basel, University of Basel, Basel, Switzerland

**Keywords:** bypass, future, history, LITA–LAD, perspective

## Abstract

Among all innovations in cardiovascular medicine, the left internal thoracic artery to left anterior descending artery (LITA–LAD) graft stands as one of the most successful and enduring therapeutic paradigms. Introduced in the late 1960s, it quickly transformed the surgical treatment of ischemic heart disease, establishing new standards of durability, patency, and survival. More than fifty years later, the LITA–LAD graft remains the cornerstone of coronary artery bypass grafting (CABG) and continues to define excellence in both cardiac surgery and interventional cardiology.

## Introduction

Among all innovations in cardiovascular medicine, the left internal thoracic artery to left anterior descending artery (LITA–LAD) graft stands as one of the most successful and enduring therapeutic paradigms. Introduced in the late 1960s, it quickly transformed the surgical treatment of ischemic heart disease, establishing new standards of durability, patency, and survival. More than fifty years later, the LITA–LAD graft remains the cornerstone of coronary artery bypass grafting (CABG) and continues to define excellence in both cardiac surgery and interventional cardiology.

## Historical relevance

The origins of coronary bypass surgery mark a defining chapter in cardiac surgery. In its early years, the saphenous vein was the preferred conduit for bypassing obstructed coronary arteries. Although easily harvested, it soon proved limited by a high rate of late occlusion due to atherosclerosis and intimal hyperplasia. The introduction of the internal thoracic artery as a conduit for the LAD changed this trajectory dramatically.

Early clinical follow-ups demonstrated superior patency and long-term survival in patients receiving a LITA–LAD graft compared with those receiving vein grafts. Namely, Lytle et al. reported excellent long-term graft durability of the internal mammary artery, with freedom from graft occlusion exceeding 90% at 10–12 years, clearly superior to saphenous vein grafts (1). Similarly, Sabik et al. demonstrated significantly greater freedom from graft occlusion of internal thoracic artery grafts across all coronary systems (2). This remarkable performance was attributed to its biological properties: resistance to atherosclerosis, preserved endothelial function, and adaptability to flow dynamics.

The LITA–LAD graft thus represents more than a surgical innovation—it is a biological success story. Its natural compatibility with the coronary circulation and its ability to function as a “living conduit” have ensured its continued superiority and widespread adoption. Few therapeutic approaches in medicine have demonstrated such sustained relevance over half a century.

## Clinical superiority and longevity

The clinical superiority of the LITA–LAD graft is evident across virtually all long-term outcome measures. In contrast to venous grafts, which may fail within a decade, the LITA maintains patency and function well beyond 20 years. Patients receiving a LITA–LAD bypass experience significantly lower rates of myocardial infarction, recurrent angina, and reoperation for graft failure (3).

When compared with other landmark therapies in cardiac surgery and cardiology, the LITA–LAD graft stands apart for its longevity and biological integration. Mechanical and bioprosthetic valves—though transformative—are subject to structural degeneration, anticoagulation-related complications, or limited durability. Likewise, percutaneous coronary interventions (PCI), despite advances in stent technology, face challenges of restenosis, thrombosis, and the need for repeat interventions. Randomized trials and meta-analyses comparing surgical revascularization with PCI in patients with proximal LAD disease have reported higher rates of repeat revascularization after PCI, even in the drug-eluting stent era, without evidence of superior long-term survival (4–6). Five year follow-up from contemporary trials indicates repeat revascularization rates of 15%–25% following PCI-DES, compared with substantially lower rates after LITA–LAD bypass (4, 5).

In contrast, a single well-functioning LITA–LAD graft can provide lifelong myocardial perfusion, representing a biologically harmonious solution rather than a prosthetic compromise. The internal thoracic artery's preserved endothelial function supports continuous nitric oxide release, maintaining vasodilation and anti-thrombotic properties. Its resistance to lipid infiltration and atherogenesis allows it to remain pristine decades after implantation, a feat unmatched by any artificial or mechanical device.

Thus, the LITA–LAD graft exemplifies the rare combination of surgical precision and biological perfection. It is not simply a successful procedure—it is a therapeutic ideal that continues to define the standards of coronary surgery.

## Foundation for modern coronary surgery of the third millennium

The influence of the LITA–LAD graft extends far beyond its historical role. It has become the biological and conceptual foundation for modern coronary surgery techniques that emphasize minimal invasiveness, precision, and patient-centered recovery.

Contemporary methods such as minimally invasive direct coronary artery bypass (MIDCAB), totally endoscopic coronary artery bypass (TECAB), and robotic-assisted CABG all rely on the proven success of the LITA–LAD construct. These procedures aim to preserve the graft's superior long-term benefits while reducing surgical trauma, avoiding sternotomy, and shortening hospital stays (4).

In MIDCAB, the graft is placed through a small anterior thoracotomy, offering targeted revascularization of the LAD territory with rapid recovery. In multivessel MIDCAB concepts LITA-LAD graft plays central role not only in the revascularization of the LAD territory but also as inflow graft for other grafts, achieving complete myocardial revascularization. TECAB takes this further by performing the entire procedure endoscopically, often assisted by robotic technology that enhances precision and visualization. Robotic CABG now allows surgeons to perform complete arterial revascularization through small ports, maintaining the biological benefits of the LITA–LAD graft while minimizing the invasiveness of the operation (7).

In every form, the LITA–LAD graft remains the centerpiece—a reliable, durable, and physiologically sound conduit around which modern surgical innovation continues to evolve. Its integration into these advanced approaches illustrates how a time-tested biological principle can coexist with cutting-edge technology to advance patient outcomes.

## Integration with interventional cardiology: the hybrid era

The emergence of hybrid coronary revascularization further underscores the enduring relevance of the LITA–LAD bypass in contemporary coronary therapy. Hybrid strategies intentionally combine the long-term durability of surgical arterial grafting with the flexibility of percutaneous coronary intervention, most commonly pairing a minimally invasive LITA–LAD bypass with PCI of non-LAD vessels.

More recent series and registries have provided quantitative clinical and angiographic data supporting this approach. Contemporary studies report early mortality rates below 1%–2%, stroke rates <1%, and LITA–LAD graft patency exceeding 95% at early and mid-term angiographic follow-up (7–10). In a comprehensive review of hybrid coronary revascularization, Moreno et al. reported freedom from major adverse cardiac events ranging from 85%–92% at 3–5 years, with repeat revascularization primarily driven by non-LAD territories rather than failure of the LITA–LAD graft (8, 9). These findings consistently identify the LITA–LAD anastomosis as the most durable component of hybrid strategies.

By leveraging the complementary strengths of surgical and percutaneous techniques, hybrid revascularization places the LITA–LAD graft as a biological anchor—providing long-term stability to the most prognostically relevant coronary territory, while PCI addresses vessels less amenable to surgical access. In this context, the LITA–LAD bypass should be regarded not as a legacy intervention, but as a living platform that continues to support the evolution of multidisciplinary, patient-tailored coronary revascularization in the third millennium.

## Future perspectives in the third millennium

Looking ahead, the future of coronary surgery will continue to be shaped by precision techniques, advanced robotics, and digital integration, supported by emerging evidence from clinical and translational research. Robotic-assisted coronary artery bypass procedures, including minimally invasive direct coronary artery bypass and totally endoscopic approaches, have demonstrated safety, feasibility, and reproducibility, with enhanced three-dimensional visualization and precise LITA harvesting compared with traditional methods (11). These technologies may reduce surgical trauma and support tailored revascularization strategies while maintaining the long-term benefits of LITA–LAD grafting.

Intraoperative assessment tools such as transit-time flow measurement have become established as functional quality control measures during coronary bypass grafting, providing real-time data on graft performance and helping to detect technical issues that might affect early graft patency (12). Integrating such objective flow mapping techniques with surgical planning and execution may further improve reproducibility and outcomes.

Emerging digital visualization tools—including dynamic four-dimensional reconstructions and mixed-reality platforms—are being developed to enhance preoperative and intraoperative spatial understanding of coronary anatomy, potentially aiding precise conduit placement and reducing intraoperative uncertainty (13). Computational modeling of coronary hemodynamics and patient-specific simulations of bypass graft performance also show promise as adjuncts for individualized surgical planning and optimization of graft configuration (14).

Concurrently, advances in vascular biology and tissue engineering are driving research into next-generation vascular conduits. Tissue-engineered vascular grafts, designed using scaffold and cell-based approaches, aim to overcome limitations in conduit availability and patency for small-diameter applications, and preclinical and clinical studies continue to explore their potential in cardiovascular surgery (15). As regenerative strategies for engineered conduits and biologically integrated grafts evolve, the internal thoracic artery remains the clinical benchmark against which these innovations are measured. Therefore, while the LITA–LAD graft represents a pinnacle of historical achievement, ongoing technological refinement and biologically inspired engineering will continue to shape the future of coronary revascularization.

## Conclusion

More than five decades after its first clinical application, the LITA–LAD graft remains one of the most successful and enduring therapies in cardiovascular medicine. Its unmatched patency, superior survival outcomes, and biological resilience have made it the benchmark for all forms of coronary revascularization.

From the early days of open-chest surgery to the present era of robotic and hybrid procedures, the LITA–LAD graft continues to define the essence of effective and durable myocardial revascularization. It is a rare example of a medical innovation whose relevance not only persists but grows stronger with time—a timeless therapeutic paradigm linking surgical tradition with technological progress and ensuring optimal outcomes for generations to come.

## Data Availability

The original contributions presented in the study are included in the article/Supplementary Material, further inquiries can be directed to the corresponding author.
